# Gut alterations in a chronic kidney disease rat model with diet‐induced vascular calcification

**DOI:** 10.1002/2211-5463.70043

**Published:** 2025-06-17

**Authors:** Piotr Bartochowski, Irene Cortijo, Shruti Bhargava, Bernard Jover, Fabrice Raynaud, Juliana H Boukhaled, Anne‐Dominique Lajoix, Vera Jankowski, Joachim Jankowski, Magali Cordaillat‐Simmons, Àngel Argilés, Nathalie Gayrard, Flore Duranton, Jonas Laget

**Affiliations:** ^1^ RD Néphrologie SAS Montpellier France; ^2^ BC2M University of Montpellier France; ^3^ Institute of Molecular Cardiovascular Research, Medical Faculty RWTH Aachen University Germany; ^4^ PhyMedExp INSERM, CNRS, University of Montpellier France; ^5^ Université Paul Sabatier Toulouse France; ^6^ Pharmabiotic Research Institute European Microbiome Regulatory Science Center Narbonne France; ^7^ UMRF Université Clermont Auvergne Aurillac France

**Keywords:** chronic kidney disease, gut, mucus, NLRP6, vascular calcification

## Abstract

Intestinal disorders and vascular calcification (VC) are often associated with chronic kidney disease (CKD). While gut barrier alterations have been reported in CKD (such as abnormal intestinal permeability, bacterial overgrowth, and inflammation), it is not clear if vascular calcification influences these alterations. To investigate whether the bidirectional relationships between VC and gut dysfunction could be mediated by increased inflammation and uremic toxin generation, we used the SNx‐VC model of uremic vascular calcification (rats undergoing subtotal 5/6th nephrectomy and fed a procalcifying high‐phosphate and vitamin D diet). We confirmed the presence of CKD and VC by von Kossa staining and observed increased gut‐origin uremic toxin, indoxyl sulfate (IS), in SNx‐VC animals compared to controls. In SNx‐VC rats, we observed decreased mucus production (Alcian blue, Mucin 2 staining) in the colon and ileum which was correlated with the level of calcification. There was no change in inflammation markers or tight junction protein expression. We assessed intestinal levels in the NOD‐like receptor family pyrin domain containing 6 (NLRP6) protein, known to regulate mucus secretion, and found no change in the colon or ileum. *Nlrp6* mRNA was, however, decreased in the colon of SNx‐VC rats, along with other mRNA (*Ly96, Sod1*), while *Tlr2* was increased compared to controls. Our observations of low mucus, low *Nlrp6* mRNA, and high IS in SNx‐VC rats confirm a link between gut barrier alterations and uremic VC. This suggests that alterations in the mucus layer could favor the generation of gut‐origin uremic toxins and promote VC in CKD. Thus, improving the gut mucus barrier function in the context of uremic VC could be considered as a possible therapeutic strategy in CKD patients.

## Graphical abstract

Chronic kidney disease (CKD) patients often suffer from intestinal and/or mineral and bone disorders. Using a rat model, we showed that uremic vascular calcification is associated with gut barrier alterations (decreased gut mucus production and *Nlrp6* gene expression, increased gut inflammation), and plasma retention of gut‐origin uremic toxins (indoxyl sulfate). Here, we propose the bone–kidney–gut axis as a target for CKD interventions.

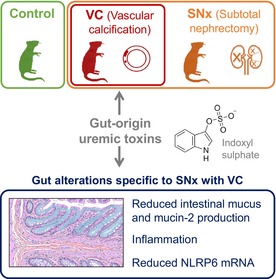

**Abbreviations**
CKD: chronic kidney disease
IS: indoxyl sulfate
NLRP6: NOD‐like receptor family pyrin domain containing 6
PCS: *p*‐cresyl sulfate
SNx: subtotal nephrectomy
VC: vascular calcification

Chronic kidney disease (CKD) is a frequent condition affecting over 10% of the global population [1]. As CKD progresses and glomerular filtration decreases, uremic solutes accumulate in the organism [2]. Pathophysiological changes occur in different organs, and alterations of the circulatory system, bone metabolism, and gastrointestinal disorders are observed [3]. In particular, patients with CKD may exhibit vascular calcification (VC) [4] that leads to impaired vessel elasticity and function (arterial stiffness) and cardiovascular events [5]. VC is characterized by the deposition of phosphate and calcium crystals (hydroxyapatite) in the arterial wall [6]. The gut, being involved in phosphate and calcium intake from diet and mineral metabolism, could affect the pathophysiology of uremic VC.

Gut microbiota is the complex ecosystem of microorganisms living in the intestine. The largest microbial community of the body is in the colon, a site of intense immune and metabolic crosstalk between the host and the microbiota [7]. The host–microbiota symbiotic relationship in the colon can be disrupted in the context of CKD with the presence of intestinal tract alterations [8]. Dialyzed patients may experience abnormal intestinal peristalsis and slowed gut transit, which could favor intestinal bacterial overgrowth [9]. Dysbiosis of gut microbiota was also observed in CKD patients [10] and numerous uremic retention solutes could originate from large intestine microbial metabolism [11]. Recently, combined microbiome metagenomics and plasma metabolomics revealed the connection between intestinal microbes and the level of circulating solutes impacted by CKD [12], such as indoxyl sulfate (IS) and *p*‐cresyl sulfate (PCS) [13], presenting a possible procalcifying effect [14]. However, few studies assessed the association between CKD, VC, and intestinal alterations. On the other hand, according to previous studies, the distal ileum is an interesting site for the observation of intestinal damages and is very susceptible to intestinal permeability, bacterial translocation, epithelial cell apoptosis [15], as well as tight junction disruption [16].

Studies in animal models of CKD indicate that the intestinal tissue also undergoes changes [17]. It was observed a reduction in the expression of tight junction proteins and a thinner mucus layer on the inner surface of the gut during CKD [18]. Inflammation of the gut in a uremic model was also demonstrated by increased immune cell infiltration and pro‐inflammatory cytokine expression [19]. Altogether, this suggests the presence of excessive intestinal permeability in CKD promoting inflammation [19, 20].

Metabolic and phenotypic changes of gut microbiota might affect the intestinal walls via the NOD‐like receptor family pyrin domain containing 6 (NLRP6). *Nlrp6* mRNA level in the gut was decreased in patients with obesity and type 2 diabetes and in rats with diet‐induced obesity [21]. But NLRP6 seems also important for kidney protection, and recently, Valiño‐Rivas *et al*. [22] showed a link between decreased NLRP6 expression and fibrosis in the kidney. However, no study has been performed on the role of NLRP6 in the intestine in CKD or CKD with VC. As an innate immune sensor that identifies molecular patterns associated with microbes, NLRP6 modulates cytokine release and toll‐like receptor expression [23]. Furthermore, NLRP6 regulates mucus secretion, as *Nlrp6* ^−/−^ mice had impaired mucus layer thickness [24], and can also affect intestinal microbiota composition to protect the intestinal epithelium [25].

In the present study, we assessed gut alterations in the ileum and colon by histological and molecular techniques in a model of uremic vascular calcification compared to uremic or control rats and evaluated the possible involvement of NLRP6.

## Methods

### Animal model

All animal experiments were performed according to the European Parliament Directive 2010/63/EU (No. CEEA‐00322.03) and approved by the local ethics committee for animal experimentation of Languedoc‐Roussillon (CEEA‐LR, no. 036, #18348). At 8 weeks of age, Sprague–Dawley rats underwent sham (Control) or subtotal nephrectomy (SNx) surgery, during which the entire right kidney and 2/3 of the contralateral kidney were removed (week 0). After 8 weeks, part of the SNx rats were fed a VC‐inducing diet enriched with phosphate (1.2% w/w chow) and vitamin D at 0.2 or 0.8 μg/day/kg body weight administered orally 5 days per week in individual pellets [26]. Twelve weeks after surgery, animals were euthanized by lethal injection of pentobarbital (200 mg·kg^−1^ I.V.) and materials were collected. Blood was collected in heparin‐containing tubes and centrifuged for 10 min at 4 °C at 1500 ***g***, after which plasma was stored at −80 °C. Tissues (kidney, thoracic aorta, colon, and ileum) were collected and sampled for calcium determination (fresh tissue), histological (formalin‐preserved), western blot (−80 °C), and qPCR (RNA protect and −80 °C) analysis.

### Biochemistry and plasma HPLC

Biological parameters were measured in blood (creatinine, urea, phosphate, calcium) and urine (creatinine, proteins) using a COBAS analyzer (Roche Diagnostics, Meylan, France). Plasma concentrations of indoxyl sulfate (IS) and *p*‐cresyl sulfate (PCS) were measured using high‐performance liquid chromatography (HPLC) and electrospray ionization mass spectrometry (ESI/MS) as previously described [27]. Briefly, 200 μL of plasma samples were deproteinized with 70% perchloric acid and centrifuged. The pH of the supernatant was adjusted to 9 with 12 m potassium hydroxide and frozen overnight at −20 °C. The next day, samples were centrifuged again to collect the supernatant, and the pH was adjusted to 7. Samples were supplemented with 0.1% formic acid to a volume of 250 μL and fractionated by HPLC with 0.1% formic acid as phase A and 100% ethanol as phase B. Fifteen fractions per sample were obtained via a stepwise gradient: Fractions 1, 2, 3: EtOH 20%; Fractions 4, 5: EtOH 40%; Fractions 6, 7, 8: EtOH 60%; Fractions 9, 10, 11: EtOH 80%; Fractions 12, 13, 14, 15: EtOH 90–100%; which were rapidly frozen and lyophilized in a vortex plate dryer. Fractions 4 and 5, where IS and PCS were detected in a pilot experiment, were dissolved together in 10 μL of 0.1% formic acid and analyzed by ESI/MS. The concentrations of IS and PCS were calculated using a calibration curve.

### Calcium content

Fresh aortic tissue samples were dehydrated at 110 °C and were decalcified with 100 μL of 0.6 m of HCl for 24 h. Calcium content was measured in the HCl supernatants using the O‐cresolphthalein complexone method according to the instructions provided by the supplier (ab102505; Abcam, Amsterdam, Netherlands) and normalized to aortic tissue dry weight.

### Histology

Pieces of kidney, aorta, colon, and ileum were fixed in formalin and embedded in paraffin. Five micrometer‐thick sections were cut and mounted on glass slides and deparaffinized, and stained. For all stains, Entellan mounting medium (Merck, Saint‐Quentin‐Fallavier, France) was applied to the slides, which were then covered with a coverslip and examined and photographed under a light microscope (Nikon Eclipse TE300, Tokyo, Japan).

For fibrosis detection, kidney tissue sections were stained with Sirius red (Sigma‐Aldrich, Saint‐Quentin‐Fallavier, France). A positive signal for collagen was seen in intense red. Kidney fibrosis was quantified with imagej software (U. S. National Institutes of Health, Bethesda, MD, USA) on 10 pictures of the renal cortex at 200‐fold magnification.

Calcium deposition in the tissue sections (aorta, colon) was assessed using von Kossa staining, which consists of treatment with silver nitrate (Sigma‐Aldrich) and counterstaining with nuclear Fast Red (Sigma‐Aldrich). Quantification of calcium deposits in aorta tissue was performed with imagej software on pictures taken from three aorta sections at 40‐fold magnification.

Alcian blue was used to detect the mucus production in ileum and colon tissue. Mucus content in the ileum and colon was also on 10 pictures of the total gut tissues at 200‐fold magnification. Results were expressed as a percentage of the tissue area stained, using imagej.

Hematoxylin/eosin was used to detect the presence of immune cells in ileum and colon tissue. Inflammatory cell infiltration in gut tissues was assessed on hematoxylin/eosin‐stained sections (10 pictures at 200‐fold magnification) using a score established by Erben *et al*. [28]. Minimal immune cell infiltration was scored 1 (< 10% of the total mucosal area), mild infiltration was 2 (10–25%), moderate infiltration was 3 (25–50%), and marked infiltration was 4 (> 50%).

### Immunostaining

CD68 positive cells and NLRP6 and Mucin 2 protein levels in the colon and ileum tissues were detected by immunohistochemistry (IHC) using anti‐CD68 (MCA341R, 1 : 200; AbD serotec, Neuried, Germany), anti‐NLRP6 (ABF29, 1 : 1000; Millipore, Molsheim, France), and anti‐mucin 2 (ab272692, 1 : 2000; Abcam) antibodies. After overnight incubation, a secondary biotinylated antibody was applied, and detection was performed with VECTASTAIN^®^ Elite^®^ ABC‐HRP Kit (PK6200; Vector Laboratories, Newark, CA, USA) and ImmPACT^®^ AEC (SK‐4205; Vector Laboratories). VectaMount™ AQ aqueous mounting medium (Vector Laboratories) was placed on the slides before they were covered with a coverslip. Slides were examined and photographed under a light microscope (Nikon Eclipse TE300). Results were expressed as a percentage of the tissue area stained using imagej.

### Western blot

Colon tissue (100 mg) from cryopreserved samples was homogenized with FastPrep^®^‐24 (MP Biomedicals, Illkirch, France) in RIPA lysis buffer (20‐188; Millipore) with the addition of a protease/phosphatase inhibitor cocktail (5872S; Cell Signaling Technology, Danvers, Massachusetts, USA). Protein concentration was estimated with Pierce BCA protein assay (23225; Thermo Scientific™, Rockford, IL, USA). Proteins (30 μg) were separated on a 10% SDS/polyacrylamide gel and transferred to a Mini PVDF membrane (1704156EDU; Bio‐Rad, Hercules, CA, USA) with a Trans‐Blot Turbo Transfer System (Bio‐Rad). After blocking in 5% milk, incubation with the first antibody was performed overnight at 4 °C. Antibodies targeting β‐actin (sc‐47778, 1 : 4000; Santa Cruz Biotechnology, Dallas, Texas, USA), Occludin (91131S, 1 : 4000; Cell Signaling Technology), and Claudin1 (ab15098, 1 : 1000; Abcam) were used as first antibodies. After incubation with appropriate secondary antibodies linked to HRP (ab6789 and ab6721; Abcam), Immobilon Western HRP substrate (WBKLS0100; Millipore) was added to the membranes. Signals were acquired with a Vilbert Lourmat imager and quantified by imagej analysis software.

### RTqPCR

Total RNA was isolated from 20 mg of RNAprotect (76106; Qiagen, Hilden, Germany) preserved samples of colonic tissue with RNeasy Mini Kit (74104; Qiagen) after homogenization with FastPrep^®^‐24 (MP Biomedical). After purification, the RNA was eluted with 50 μL RNAse‐free water. RNA quality and quantity were checked with 1.6% electrophoresis gel and NanoDrop™ One instrument (Thermo Scientific™). First strand cDNA was synthesized, from 1 mg RNA, at 37 °C for 60 min with Omniscript^®^ Reverse Transcription kit (205111; Qiagen). Specific primers listed in Fig. S1 were designed with lightcycler Probe Design Software 2.0 (version 1.0.R.36; Roche) from mRNA sequences found in the NCBI nucleotide database and validated experimentally. Primers were synthesized by Integrated DNA Technologies (Leuven, Belgium). The quantitative PCR was performed using 2.5 ng cDNA, 500 nm forward and reverse primers, and LightCycler 480 SYBR Green I Master kit (Roche) in the LightCycler^®^ 96 Instrument (Roche). Genetic expression was quantified as the ratio of the expression of the mRNAs of interest normalized to the expression of three reference genes: *Beta‐actin (β‐actin)*, *Hypoxanthine phosphoribosyltransferase 1 (Hprt1)*, and *Ribosomal protein L32 (L32)*.

### Statistical analysis

For continuous variables, we assessed normality and performed logarithmic transformation or rank transformation (for calcium content, phosphatemia, NLRP6 staining in colon) to reach normality. Statistical tests were performed on transformed variables, and results are presented in the original scale. Differences in means between groups were analyzed by ANOVA followed by Tukey–Kramer's test for pairwise comparisons. One‐sample *t*‐tests were used to compare a group means to a specific value. For categorical variables, differences between groups were tested by Fisher's exact tests. Pearson correlations were performed between quantitative variables, and *P*‐values were adjusted to a false discovery rate of 5% using the Benjamini–Hochberg method. All tests considered a bilateral 5% type 1 error. All data are reported as mean ± SEM, unless stated otherwise.

## Results

### Model characteristics

According to the protocol, rats were grouped as Control (sham surgery), SNx, which underwent subtotal nephrectomy, and SNx‐VC, which had subtotal nephrectomy followed by a high‐phosphate and vitamin D diet to induce VC. At sacrifice, the SNx and SNx‐VC groups had higher plasma levels of creatinine and doubling kidney fibrosis, which indicate lower renal function and integrity (Table 1 and Fig. S1a). Plasma phosphate, calcium, and Ca × P product were increased in the SNx‐VC group (Table 1). Calcium content in the thoracic aorta was increased in the SNx‐VC animals compared to the Control and SNx groups (*P* < 0.05, Table 1). Aortic calcifications were observed in the SNx‐VC group only, in 67% of animals (Table 1). The positive von Kossa staining of aorta sections was 10.2% ± 2.9 of the surface area in the SNx‐VC group and 15.3% ± 3.0 when limiting to animals presenting VC (Table 1 and Fig. S1b). One animal with aortic calcifications also displayed calcified vessels in the gut (Fig. S1c).

**Table 1:** Biochemical and histological markers of CKD and VC by group.

	Control (*n* = 6)	SNx (*n* = 6)	SNx‐VC (*n* = 12)	*P*‐value
Plasma				
Creatininemia^a^ (μm)	25.67 ± 1.31	91.20 ± 19.62^b^	99.17 ± 18.35^b^	0.0002
Urea^a^ (mm)	5.60 ± 0.18	14.40 ± 2.89^b^	13.09 ± 1.72^b^	0.001
Phosphatemia^a^ (mm)	1.79 ± 0.03	2.11 ± 0.11	2.71 ± 0.26^b^	0.001
Calcemia (mm)	2.51 ± 0.02	2.72 ± 0.04	2.81 ± 0.06^b^	0.02
Ca × P product^a^ (mm ^2^)	4.50 ± 0.12	5.73 ± 0.32	7.59 ± 0.69^b^	0.004
IS^a^ (μg·L^−1^)	1455 ± 257	5024 ± 1252^b^	6482 ± 1414^b^	0.002
PCS^a^ (μg·L^−1^)	147 ± 65	266 ± 88	1511 ± 755	0.13
Urine protein (g/L)	0.50 ± 0.09	2.44 ± 0.57^b^	2.14 ± 0.35^b^	0.0002
Kidney fibrosis^a^ (positive area %)	4.74 ± 0.7	9.1 ± 1.7	10.4 ± 1.2	0.054
Thoracic aorta				
Calcium content^a^ (nmol per 100 mg)	20.0 ± 1.8	19.1 ± 1.1	1211.2 ± 347.4^b,c^	0.01
Calcified surface area (% positive area)	0 ± 0	0 ± 0	10.2 ± 2.9^b,c^	0.005
Calcified thoracic aorta (cases (%))	0 (0%)	0 (0%)	8 (67%)^b,c^	0.01
Calcium content^a^ (nmol per 100 mg)	–	–	1801.3 ± 367.7	–
Calcified surface area (% positive area)	–	–	15.3 ± 3.0	–

^a^ Variable transformed for statistical analysis

^b^ *P*‐value < 0.05 compared to control; ^c^ *P*‐value < 0.05 compared to SNx, from Tukey *post hoc* tests after ANOVA.

**Fig. 1:**
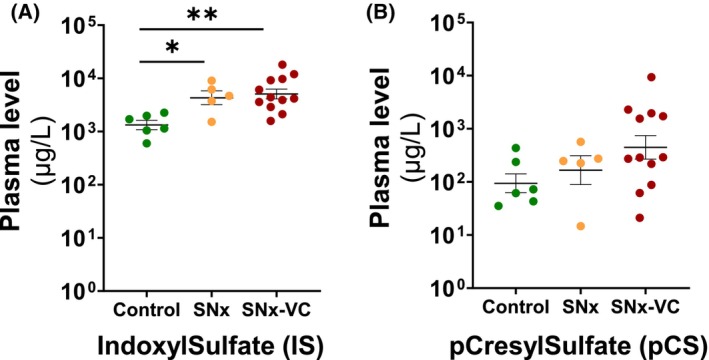
Determination of plasma concentrations of gut‐related uremic toxins in the different groups of rats: the level of indoxyl sulfate increased in the plasma of SNx rats with or without VC, while the level of *p*‐cresyl sulfate remained statistically unchanged. (A) Plasma concentration of IS and (B) Plasma concentration of PCS measured by ESI/MS. Data are presented as mean ± SEM, and each point represents data from an individual rat. *P*‐value from Tukey *post hoc* tests after ANOVA are shown: *P < 0.05; **P < 0.01.

Compared to Control, IS was increased in SNx (*P* < 0.05) and SNx‐VC (*P* < 0.01) in a similar manner (Table 1, Fig. 1). Plasma PCS levels were more variable, and group means were not significantly different.

### Tissue alterations in the colon and ileum

**Fig. 2:**
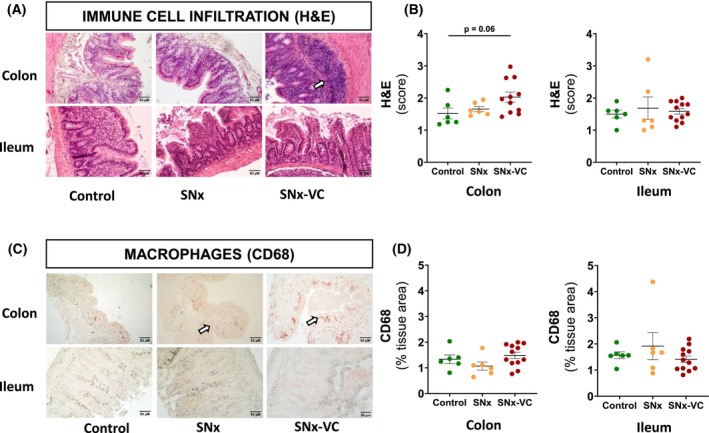
Colon and ileum tissues show no clear infiltration of immune cells in SNx rats with or without VC compared with controls. (A) Representative hematoxylin/eosin‐stained colon and ileum sections. The arrow indicates the presence of an accumulation of immune cells and highlights cellular infiltration. Original magnification ×200. (B) Immune cell infiltration score was quantified using hematoxylin/eosin staining according to Erben *et al*. [28]. (C) Representative CD68 immunostaining of colon and ileum sections showing CD68 positive cells (arrow). Original magnification ×200. (D) CD68‐positive cells quantification (percentage of tissue stained) by immunohistochemistry (IHC). Original magnification ×200. Data are presented as mean ± SEM, and each point represents data from an individual rat. *P*‐value < 0.1 from Tukey *post hoc* tests after ANOVA is shown.

Hematoxylin/eosin staining of gut showed an accumulation of small dark cells in mucosal tissue (Fig. 2A). The scoring of immune cell infiltration did not show differences between groups for ileum (*P* = 0.8, Fig. 2A,B) and was increased in SNx‐VC in colon (*P* = 0.06, Fig. 2A,B). CD68‐positive cells were mainly located in the outer parts of the mucosal folds, bordering the intestinal lumen (see arrow in Fig. 2C). There was no difference between groups in the percentage of area stained for CD68 in colon or ileum (Fig. 2D).

**Fig. 3:**
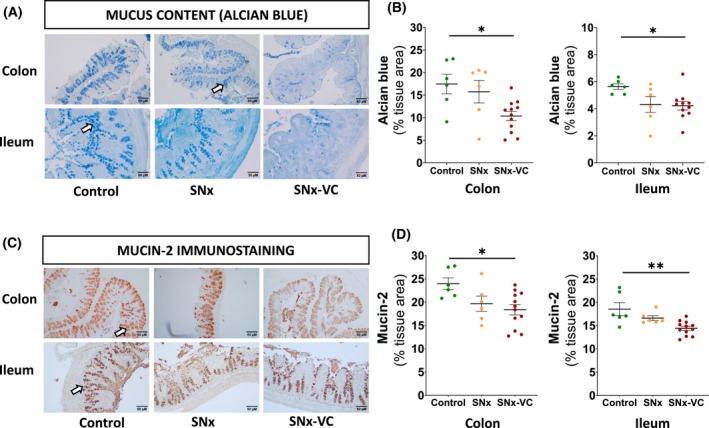
Mucus content and Mucin 2 level decreased in SNx rats with vascular calcification compared to control rats in colon and ileum tissues. (A) Representative alcian blue staining of colon and ileum sections. Original magnification ×200. (B) Mucus content quantification using alcian blue staining. (C) Representative Mucin 2 immunostaining in colon and ileum sections. Original magnification ×200. (D) Mucin 2 protein level by IHC. Arrows indicate goblet cells. Data are presented as mean ± SEM, and each point represents data from an individual rat. *P*‐value from Tukey *post hoc* tests after ANOVA are shown: *P < 0.05; **P < 0.01.

Mucus content was evaluated with Alcian blue stain and immunostaining of Mucin 2. Positive stains were located in goblet cells, where secretory vesicles containing mucus are present (Fig. 3A,C). Alcian blue stain indicated that mucus content was decreased in the colon (−7.1% of total area stained, *P* = 0.017) and ileum (−1.4%, *P* = 0.04) of SNx‐VC rats versus Control (Fig. 3B). Mucin 2 protein expression was similarly decreased in the colon (*P* = 0.02) and ileum (*P* = 0.002) of SNx‐VC rats (Fig. 3D). Moreover, CD68‐positive area was inversely correlated with Alcian blue staining of mucus (*r* = −0.47) and with Mucin 2 protein (IHC, *r* = −0.62) in the colon (see Fig. S2).

### Gene expression in colon

We investigated mRNA expression of genes involved in mucus production (*Mucin 2*), immunity and inflammation (*Il6*, *Il10*, *Il18*, *NFκB*, *Ccl2/Mcp1*, *Tlr2, Tlr4, Ly96/MD‐2, Aoah, Nlrp6*), tight junction (*Cldn1, Cldn2, Cldn4, Ocln, Tjp1/ZO‐1*), and antioxidant activity (*Sod1, Cat*) in colon (Table 2, see Table S1 for abbreviation meaning). No significant change between groups was detected by ANOVA. However, using one‐sample *t*‐tests, we identified a significant decrease in *Nlrp6*, *Ly96*, and *Sod1* in SNx‐VC compared to the Control group (mean of 1 as null hypothesis), and an increase in *Tlr2* expression (Table 2). We also found increased expression of *Il18* in SNx versus Control rats.

**Table 2:** Relative mRNA expression in the colon normalized to housekeeping genes (*β‐actin/Hprt1/L32*) expressed as ratio of the control group and correlation with *Nlrp6* mRNA. Relative mean ± SEM.

Name	Gene	Mean value relative to control		Correlation with *Nlrp6* gene expression	
		Snx (*n* = 6)	Snx‐VC (*n* = 6)	*r*	*P*‐value^a^
NOD‐like receptor pyrin domain containing 6	*Nlrp6*	0.74 ± 0.17	**0.55 ± 0.17** ^b^	1	–
Claudin‐2	*Cldn2*	0.91 ± 0.21	0.75 ± 0.15	**0.72**	**0.009**
Lymphocyte antigen 96	*Ly96 (MD‐2)*	0.84 ± 0.22	**0.58 ± 0.10** ^b^	**0.71**	**0.009**
Tight junction protein ZO‐1	*TJP1 (ZO‐1)*	1.23 ± 0.27	0.88 ± 0.14	**0.66**	**0.017**
Superoxide dismutase 1	*Sod1*	1.05 ± 0.13	**0.68 ± 0.07** ^b^	**0.64**	**0.017**
Nuclear factor kappa B subunit 1	*NFκB*	1.14 ± 0.14	1.07 ± 0.07	**0.62**	**0.020**
Catalase	*Cat*	1.16 ± 0.43	1.20 ± 0.62	**0.61**	**0.020**
Occludin	*Ocln*	1.02 ± 0.12	0.86 ± 0.12	**0.60**	**0.024**
Claudin‐4	*Cldn4*	1.30 ± 0.29	1.31 ± 0.52	0.23	0.61
Mucin 2	*Muc2*	0.62 ± 0.17	0.85 ± 0.14	0.14	0.61
C‐C motif chemokine ligand 2	*Ccl2 (Mcp1)*	1.21 ± 0.30	1.18 ± 0.28	0.12	0.65
Interleukin 18	*Il18*	**1.59 ± 0.23** ^b^	2.16 ± 0.68	0.06	0.81
Interleukin 10	*Il10*	3.79 ± 2.84	1.40 ± 0.21	0.03	0.84
Interleukin 6	*Il6*	3.01 ± 2.05	1.23 ± 0.12	0.01	0.93
Acyloxyacyl hydrolase	*Aoah*	1.42 ± 0.61	1.39 ± 0.32	−0.06	0.93
Claudin‐1	*Cldn1*	0.69 ± 0.15	1.25 ± 0.34	−0.22	0.96
Toll‐like receptor 2	*Tlr2*	0.99 ± 0.13	**1.31 ± 0.11** ^b^	−0.25	0.96
Toll‐like receptor 4	*Tlr4*	0.72 ± 0.10	1.29 ± 0.27	−0.49	0.10

*Note*: Bold values associated with a *P*‐value < 0.05.

^a^ Pearson correlation *P*‐values adjusted for a false discovery rate (FDR) of 5% by Benjamini–Hochberg method

^b^ *P*‐value < 0.05 from one‐sample *t*‐test vs. 1.

We observed positive correlations between *Nlrp6* mRNA expression and *Cldn2, Tjp1, Ocln, Sod1, Cat, Ly96*, and *NFκB* mRNA (all *P*‐value < 0.01, Table 2, Fig. S2). *Nlrp6* mRNA expression was negatively correlated with *Tlr4* (*P* < 0.05, Table 2).

### Protein expression

**Fig. 4:**
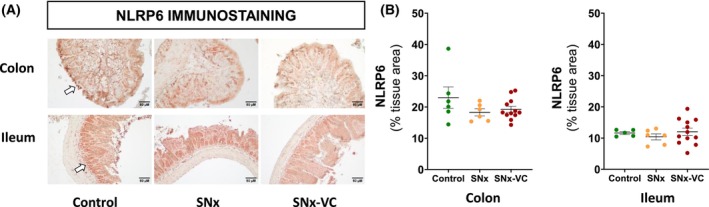
The level of NLRP6 protein is not altered by CKD in the colon and ileum. (A) Representative images of NLRP6 staining in colon and ileum. Arrows show mucosal tissue. Magnification ×200. (B) NLRP6 protein level by IHC. Data are presented as mean ± SEM, and each point represents data from an individual rat.

We assessed NLRP6 protein expression in the gut as a potential regulator of mucus production, inflammation, and gut integrity. Immunostaining showed that NLRP6 protein was mainly located in mucosal tissue and aggregated in the peripheral area adjacent to the intestinal lumen and intestinal glands (Fig. 4A). There was no difference in NLRP6 immunostaining between groups in the colon (*P* = 0.23) or ileum (*P* = 0.62) (Fig. 4B).

**Fig. 5:**
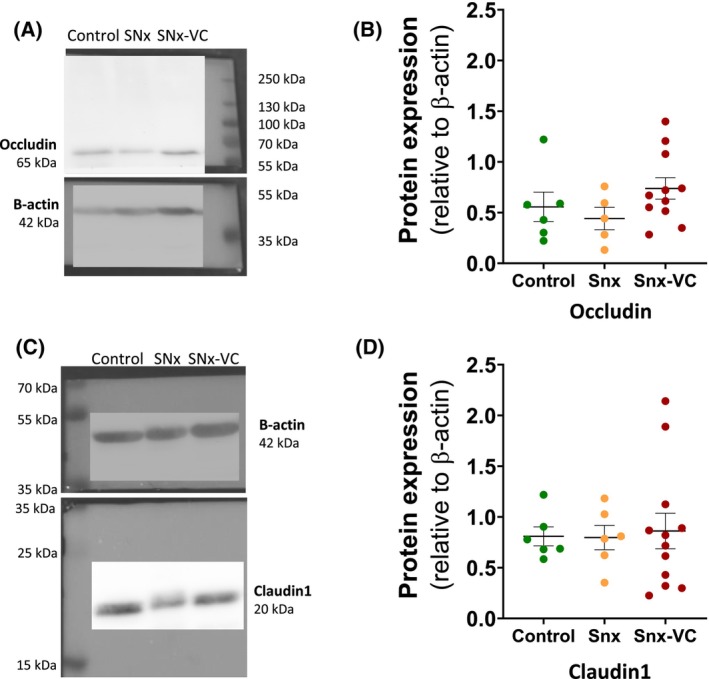
Occludin and Claudin 1 protein levels are unchanged in colon tissue of SNx rats compared to control rats. (A) Representative image of Occludin and β‐actin expressions in colon tissue by western blot. (B) Quantification of Occludin protein expression by western blot (expressed as a ratio to β‐actin expression). (C) Representative image of Claudin1 and β‐actin expressions in colon tissue by western blot. (D) Quantification of Claudin1 protein expression by western blot (expressed as a ratio to β‐actin expression). Data are presented as mean ± SEM, and each point represents data from an individual rat.

We investigated the expression of the tight junction protein Occludin and Claudin‐1 in colon by western blot (Fig. 5A,C). Observed protein levels were unchanged between groups and did not indicate the presence of epithelial barrier disruption (Fig. 5B,D).

### Correlations between markers of CKD, VC, and gut alterations

We explored interesting correlations in uremic rats (*n* = 18, Table 3). Overall, kidney fibrosis was highly correlated with CKD (plasma urea and creatinine, *r* ≥ 0.8) and phosphate (*r* = 0.78) and significantly, yet less importantly, with VC (Aorta VC, *r* = 0.62). It was negatively associated with *Ocln*, *Il18*, and *Nlrp6* mRNA expression in the colon. The correlation pattern of plasma IS was very similar to the one of kidney fibrosis (Table 3). In addition, a remarkable negative correlation was observed between mucus production in the colon and circulating PCS (*r* = −0.56) (see Fig. S2).

**Table 3:** Correlation heatmap limited to significant associations with kidney fibrosis, plasma IS and/or aortic calcium content in uremic rats (SNx and SNx‐VC).

	Kidney fibrosis	Aorta calcium content	Plasma IS
Kidney fibrosis	**1.00**	0.53	**0.75**
Plasma urea	**0.82**	0.31	**0.82**
Plasma creatinine	**0.80**	0.42	**0.86**
Plasma Phosphate	**0.78**	**0.79**	**0.67**
Plasma PxCa	**0.73**	**0.75**	**0.70**
Plasma IS	**0.75**	0.56	**1.00**
Plasma PCS	**0.69**	**0.73**	**0.89**
Aorta VC	**0.62**	**0.78**	**0.69**
Colon mucus	−0.08	**−0.60**	−0.33
Ileum mucin 2	−0.28	**−0.67**	−0.17
*Sod1* mRNA	−0.26	**−0.77**	−0.30
*Ocln* mRNA	**−0.72**	−0.41	−0.61
*Il18* mRNA	**−0.63**	0.12	−0.46
*Nlrp6* mRNA	**−0.65**	−0.32	−0.47

*Note*: Bold indicates, Pearson correlation coefficients with FDR‐adjusted *P*‐value < 0.05. A color gradient represents correlation coefficient values from low (dark blue) to high (dark red).

The level of aortic calcium content showed a slightly different correlation pattern. It was tightly correlated with other markers of VC (P, von Kossa) and with plasma IS and PCS levels but did not correlate significantly with plasma urea and creatinine (*r* < 0.42). Interestingly, aortic calcium content was negatively correlated with colon mucus (Alcian blue staining), ileum Mucin 2 protein (immunostaining), and *Sod1* mRNA (Table 3).

A correlation heatmap for all variables is available in Fig. S2. At mRNA level in colon, *Mucin 2* expression was inversely correlated with *Il6* (*r* = −0.62) and *Il10* (*r* = −0.59). *Ccl2* expression was strongly correlated with plasma creatinine (*r* = 0.81) and IS level (*r* = 0.55) (Fig. S2). Interestingly, *Nlrp6* mRNA expression in colon correlated negatively with kidney fibrosis (*r* = −0.65) and plasma IS (*r* = −0.47) and positively with the mRNA expression of *Ly96* (*r* = 0.76), *NFκB* (*r* = 0.59), *Claudin 2* (*r* = 0.69), *Tjp1* (*r* = 0.66), *Occludin* (*r* = 0.62), *Catalase* (*r* = 0.68) and *Sod1* (*r* = 0.49).

## Discussion

The present study focused on gut barrier alterations in a recently established rodent model of uremic VC. Our main finding was a decreased mucus production in the colon and ileum of calcified rats, correlated with calcification severity. Mucus layer alteration in these animals could have promoted the absorption of gut‐origin precursors of uremic toxins, such as PCS and IS. Indeed, PCS and IS circulating levels negatively correlated with colonic mucus production. In the colon, *Mucin 2* mRNA expression was inversely correlated to the cytokines *Il6* and *Il10*, illustrating possible consequences of mucus barrier dysfunction for the inflammatory response. In addition, *Nlrp6* mRNA expression in the colon was negatively correlated with kidney fibrosis and circulating IS and correlated with genes involved in tight junction, inflammation, and oxidative stress regulation. At the protein level, NLRP6 was not decreased in the gut in CKD rats.

We developed a surgical and diet‐induced rodent model of medial VC, while limiting stressful interventions such as oral gavage or intraperitoneal injections and allowed sufficient time for CKD to progress before inducing calcification [26]. There was no indication of major diet‐induced toxicity (i.e., pellet aversion, mortality) and the doses of α‐calcidol (0.2 or 0.8 μg·kg^−1^) resulted in similar levels of VC, but the incidence increased with the higher dose.

Mucus barrier is crucial to prevent translocation of commensal and pathogenic bacteria to the host, and gut mucus is mainly composed of Mucin 2 produced by goblet cells [29]. Mucus also maintains healthy gut microbiota by providing complex carbohydrates (i.e., glycans) that will support the growth of specific bacteria [30]. Colon has a two‐layered mucus with inner layer impenetrable to bacteria [31, 32]. The penetration of bacteria and their contact with epithelium was observed in murine colitis models and patients with ulcerative colitis [33]. In the present study, we assessed mucus content in goblet cells, with Alcian blue stain and Mucin 2‐targeted IHC. In calcified uremic rats, a decreased mucus production was observed in both ileum and colon as well as a negative correlation between mucus production and vascular calcification severity. Hence, our results support the ones from Yan *et al*. [34] that described a reduction of goblet cells number and Mucin 2 staining in the colon of vitamin D plus nicotine (VDN) rat, another model of vitamin D‐induced VC [35, 36]. Decreased mucus production was also previously observed in a rat model of CKD [18] but data in CKD patients have not been reported yet [17]. At mRNA level, a negative correlation was observed in the expression of *Mucin 2* versus the pro‐inflammatory cytokine *Il6*. This is in line with the local low expression of Mucin 2 associated with a high expression of Il6 that was previously observed in colon cancer [37].

In calcified SNx‐VC rats, the presence of inflammatory cells increased in the colon. Colonic macrophage infiltration (CD68) did not differ in CKD rats compared to Control, in line with the results of Yang *et al*. [38], but macrophage presence was inversely correlated to mucus production. We observed high IS levels in the plasma of SNx and SNx‐VC rats, and plasma PCS negatively correlated with mucus production in the colon. Thus, we hypothesized that a thinner mucus layer in SNx‐VC rats favored the absorption of gut‐origin metabolites. Indeed, if the colonic mucus layer is thinner, bacteria are closer to the gut epithelium, and the exposure to bacterial products increases. Furthermore, mucus thickness and physicochemical properties (pore size, viscoelasticity, ionic strength, pH, …) affect permeability to molecules [39, 40] which could influence indole and *p*‐cresol absorption and consequently plasma IS and PCS levels [41]. Nonetheless, the increased level of circulating gut‐origin uremic toxins in SNx rats is also the consequence of decreased kidney function. Indeed, in CKD it can be challenging to determine if the increased blood level of a given uremic toxin is primarily due to decreased elimination by the kidneys or increased intestinal production and absorption [11, 42]. However, it is known that serum IS concentration is associated with VC presence in CKD patients [43] and IS administration in hypertensive rats leads to aortic calcification [44]. In the present study, IS level and VC in the thoracic aorta correlated, and we may assume that IS participates in the establishment of VC in SNx‐VC rats, as suggested by previous work [44].

Analysis of colonic mRNA expression shows that most of the targeted genes were not differentially expressed between groups. Still, changes in the expression of mRNA involved in inflammatory processes were observed, such as decreased expression of *Nlrp6* and *Ly96/MD‐2*, co‐receptor of TLR4 for bacterial lipopolysaccharides [45, 46], as well as increased expression of *Tlr2*, in SNx‐VC group. Furthermore, the pro‐inflammatory cytokine *Il18* [46, 47] was increased in the uremic rats, significantly in the SNx group. In calcified CKD rats (SNx‐VC group), there was a decreased *Sod1* mRNA expression, a potential sign of oxidative stress, which may have amplified pro‐inflammatory processes [48]. Nevertheless, Claudin‐1 and Occludin protein expression evaluated by western blot in the colon was unchanged, and our data do not support the presence of tight junction disturbance in the model.

Finally, *Nlrp6* mRNA expression in the colon was highly correlated with genes involved in tight junction (*Claudin‐2, Tjp1/ZO‐1, Occludin*), inflammation (*Ly96/MD‐2, NFκB*), and oxidative stress regulation (*SOD1, Catalase*). Colon *Nlrp6* mRNA level was also negatively associated with kidney fibrosis (Sirius red) and plasmatic IS level. Wlodarska *et al*. [24] showed that the NLRP6 inflammasome was expressed in colonic goblet cells where it regulates mucus secretion, which was confirmed by Birchenough *et al*. [49]. NLRP6 was recently shown to protect from intestinal damage in patients and rodents with obesity and type 2 diabetes [21]. Nevertheless, the protein level and gene expression of NLRP6 were similar between the groups in the present study, which could be due to a lack of technical sensitivity or alternatively to increased protein translation efficacy or protein stability. Still, the reduced *Nlrp6* mRNA expression in SNx‐VC was associated with decreased mRNA expression of tight junction, inflammatory, and antioxidant proteins, which indicates that *Nlrp6* could be an important regulator of colon adaptation in this model. The role of NLRP6 in the changes observed in the lower gastrointestinal tract during CKD and VC still requires confirmation.

The main limitation of our study is that we found intermediate results in the SNx group, which could be due to a lack of power or the absence of effect in these animals. Still, we identified significant changes in the SNX‐VC group, which had similar kidney fibrosis compared to SNx. The design used, with high‐phosphate and vitamin D in the diet, could have directly affected gut microbiota, which was not evaluated here. Another limitation of the study is that we did not test the effect of the VC‐inducing diet in control animals. While we do not expect vascular calcification to occur in the absence of renal mass reduction, this would have enabled to assess the effect of the high‐phosphate and vitamin D diet on gut tissue. However, results from Yan *et al*. [34] in a nondiet‐based VC model confirm and strengthen our observations of a link between mucus, IS, and VC. Finally, we did not evaluate the effect of pharmacological or nutritional interventions, which would have helped in deciphering the mechanisms involved in the development of CKD and VC‐related gut alterations. In particular, short‐chain fatty acids have been shown to affect mucus production and, more recently, vascular calcification, making them interesting candidates to explain the modifications observed in CKD‐associated VC [18, 34].

## Conclusions

We observed significant modifications in gut structure and function associated with our model of CKD and vascular calcification. Even if we cannot conclude causation, the results presented here suggest that improving the gut mucus barrier function in the context of uremic VC could be considered a possible therapeutic strategy to try to limit gut‐origin uremic toxin level, local inflammation, and calcification in CKD patients.

## Conflict of interest

The authors declare no conflict of interest.

## Peer review

The peer review history for this article is available at https://www.webofscience.com/api/gateway/wos/peer‐review/10.1002/2211‐5463.70043.

## Author contributions

PB contributed to conceptualization, data curation, formal analysis, investigation, methodology, visualization, and writing—original draft. IC contributed to data curation, investigation, and methodology. SB contributed to data curation, investigation, and methodology. BJ contributed to conceptualization, investigation, and methodology. FR contributed to investigation and methodology. JB contributed to data curation and investigation. A‐DL contributed to funding acquisition, project administration, and supervision. VJ contributed to data curation, methodology, and resources. JJ contributed to data curation, methodology, and resources. MC‐S contributed to conceptualization, methodology, and supervision. ÀA contributed to conceptualization, funding acquisition, methodology, project administration, and supervision. NG contributed to conceptualization, data curation, investigation, funding acquisition, methodology, project administration, and supervision. FD contributed to formal analysis, visualization, project administration, supervision, and writing—review and editing. JL contributed to conceptualization, data curation, formal analysis, investigation, methodology, supervision, and writing—review and editing. All authors have read and agreed to the published version of the manuscript.

## Supporting information


**Fig. S1.** Representative images of kidney fibrosis, thoracic aorta calcification and colonic calcification.
**Fig. S2.** Correlation heatmap for all variables in uremic rats.
**Table S1.** qPCR primers.

## Data Availability

Data underlying this study is available under a CC0 license in Dryad at https://doi.org/10.5061/dryad.k0p2ngfhx.
