# Supplementary material for: Gut alterations in a chronic kidney disease rat model with diet‐induced vascular calcification
Source: FEBS Open Bio. 2025 Jun 17;15(8):1219–31. doi: 10.1002/2211-5463.70043 (PMC12319703; doi:10.1002/2211-5463.70043)

Gut alterations in a chronic kidney disease rat model with diet-induced vascular calcification

Piotr Bartochowski, Irene Cortijo, Shruti Bhargava, Bernard Jover, Fabrice Raynaud, Juliana Boukhaled, Anne-Dominique Lajoix, Vera Jankowski, Joachim Jankowski, Magali Cordaillat-Simmons, Àngel Argilés, Nathalie Gayrard, Flore Duranton and Jonas Laget

**Supporting information**

**Supplementary Table. qPCR primers**

List of the Forward and Reverse primers used for the qPCR analysis

**Supplementary Figure 1. Representative images of kidney fibrosis, thoracic aorta calcification and colonic calcification**(a) Sirius red staining of renal tissue. Original magnification x200.

(b) Von Kossa staining of the thoracic aorta. Mosaic image from observations at magnification x40

(c) Von Kossa of intestinal vessels. Black-framed area in the photo on the left (magnification x40) shown magnified on the right (magnification x200).

**Supplementary Figure 2. Correlation heatmap for all variables in uremic rats.**

Pearson correlations were performed in SNx and SNx-VC rats on transformed variables.

**Supplementary Table. RTqPCR primers**

List of the Forward and Reverse primers used for the qPCR analysis

| **Gene Category** | **Gene Symbol** | **Gene name** | **Primer direction** | **Sequence** | **Amplicon size (bp)** |
| --- | --- | --- | --- | --- | --- |
| **Housekeeping** | *β-actin* | *Beta-actin* | For | GATCAAGATCATTGCTCCTCCTG | 183 |
|  |  |  | Rev | AGGGTGTAAAACGCAGCTCA |  |
|  | *Hprt1* | *Hypoxanthine phosphoribosyltransferase 1* | For | TCCTCCTCAGACCGCTTTTC | 78 |
|  |  |  | Rev | ATCACTAATCACGACGCTGGG |  |
|  | *L32* | *Ribosomal protein L32* | For | GAAAACCAAGCACATGCTGC | 87 |
|  |  |  | Rev | TTGTTGCACATCAGCAGCAC |  |
| **Mucus** | *Muc2* | *Mucin 2* | For | ACCACCATTACCACCACCTCAG | 125 |
|  |  |  | Rev | CGATCACCACCATTGCCATTG |  |
| **Inflammation** | *Il6* | *Interleukin 6* | For | AAGTTAGAGTCACAGAAGGAG | 111 |
|  |  |  | Rev | ACACTAGGTTTGCCGAG |  |
|  | *Il10* | *Interleukin 10* | For | CTGCTATGTTGCCTGCTCTTA | 82 |
|  |  |  | Rev | AGTGGGTGCAGTTATTGTCA |  |
|  | *Il18* | *Interleukin 18* | For | GACCGAACAGCCAACGAATC | 85 |
|  |  |  | Rev | ATAGGGTCACAGCCAGTCCT |  |
|  | *Ccl2 (Mcp1)* | *Chemokine (C-C motif) ligand 2* | For | TGTCTCAGCCAGATGCAGTT | 80 |
|  |  |  | Rev | CAGCCGACTCATTGGGATCA |  |
|  | *Aoah* | *Acyloxyacyl hydrolase* | For | CCTGTAATGGCTGGATGTC | 80 |
|  |  |  | Rev | CAGTATGTTGGAGAGTTGTTC |  |
|  | *Cat* | *Catalase* | For | CACTTTGACAGAGAGCGG | 103 |
|  |  |  | Rev | CCTTGGAGTATCTGGTAATATCG |  |
|  | *Sod1* | *Superoxide dismutase 1* | For | CAATGTGTCCATTGAAGATCG | 100 |
|  |  |  | Rev | TTTGCCCAAGTCATCTTGTT |  |
| **Sensing** | *Nlrp6* | *Nod‐like receptor family pyrin domain containing 6* | For | GACACTCAGGATACAGATGC | 96 |
|  |  |  | Rev | CTGAGGTCCAGAGTGGTT |  |
|  | *Tlr2* | *Toll-like receptor 2* | For | TTGCAGGGACCTTTGCTA | 81 |
|  |  |  | Rev | TGCTGGACCATGAGGTT |  |
|  | *Tlr4* | *Toll-like receptor 4* | For | CTCTGCCCTGCCACCATTTA | 169 |
|  |  |  | Rev | AGGAAGTACCTCTATGCAGGGAT |  |
|  | *Ly96 (MD-2)* | *Lymphocyte antigen 96* | For | GGAACCAATGGATTTGTGC | 115 |
|  |  |  | Rev | CAATTTCCTTGCGCTTCG |  |
|  | *Nf-кb* | *Nuclear factor kappa-light-chain-enhancer of activated B cells* | For | GACTTCTCCTCCATTGCG | 83 |
|  |  |  | Rev | TGAGCAGGGTCACCATC |  |
| **Tight junction** | *Ocln* | *Occludin* | For | CACATCAAGAGGATGGTGG | 82 |
|  |  |  | Rev | GCACCTCTCTCTCACTTC |  |
|  | *Cldn1* | *Claudin-1* | For | GGTAGACCTGGATTTGCAT | 82 |
|  |  |  | Rev | GCAATACATTTAGGGCAATCG |  |
|  | *Cldn2* | *Claudin-2* | For | CTGACCTGGAAGCCATTT | 91 |
|  |  |  | Rev | GGGCAGGAAGGAGTGATTA |  |
|  | *Cldn4* | *Claudin-4* | For | GACTCCTCCCGGTGATT | 80 |
|  |  |  | Rev | TCCAGCCACTCCACAGAA |  |
|  | *Tjp1 (ZO-1)* | *Tight junction protein ZO-1* | For | AGTTTGACAGTGGAGTCG | 93 |
|  |  |  | Rev | CAGGAACAGCTTTAGGCAT |  |

**Supplementary Figure 1. Representative images of kidney fibrosis, thoracic aorta calcification and colonic calcification**

(a) Sirius red staining of renal tissue. Original magnification x200. (b) Von Kossa staining of the thoracic aorta. Mosaic image from observations at magnification x40. (c) Von Kossa of intestinal vessels. Black-framed area in the photo on the left (magnification x40) shown magnified on the right (magnification x200).

**a)**

**c)**

**b)**

**Supplementary Figure 2. Correlation heatmap for all variables in uremic rats.**

Pearson correlations were performed in SNx and SNx-VC rats on transformed variables.


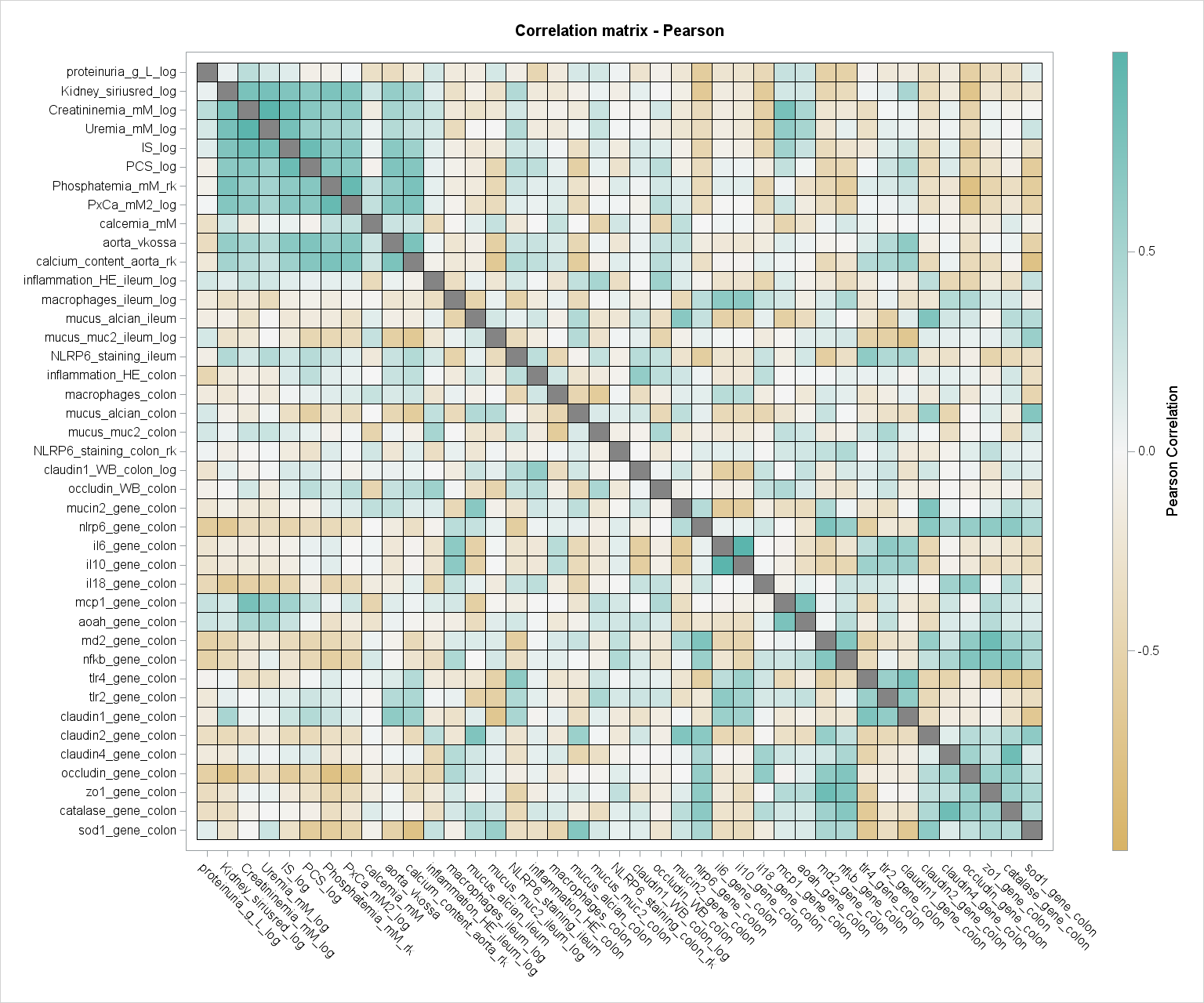

Supplement: Supplementary file 1 — Fig. S1. Representative images of kidney fibrosis, thoracic aorta calcification and colonic calcification. Fig. S2. Correlation heatmap for all variables in uremic rats. Table S1. qPCR primers. [file FEB4-15-1219-s001.docx]
